# Association of glucose-6-phosphate dehydrogenase deficiency and malaria: a systematic review and meta-analysis

**DOI:** 10.1038/srep45963

**Published:** 2017-04-06

**Authors:** Evaristus Chibunna Mbanefo, Ali Mahmoud Ahmed, Afaf Titouna, Ahmed Elmaraezy, Nguyen Thi Huyen Trang, Nguyen Phuoc Long, Nguyen Hoang Anh, Tran Diem Nghi, Bui The Hung, Mai Van Hieu, Nguyen Ky Anh, Nguyen Tien Huy, Kenji Hirayama

**Affiliations:** 1 Department of Immunogenetics, Institute of Tropical Medicine (NEKKEN), Nagasaki University, Sakamoto, Nagasaki, Japan; 2 Department of Parasitology and Entomology, Nnamdi Azikiwe University, P.M.B. Awka, Nigeria; 3 Faculty of Medicine, Al-Azhar University, Cairo, Egypt; 4 University of Medicine and Pharmacy at Hue City, Hue City, Vietnam; 5 University of Medicine and Pharmacy at Ho Chi Minh City, Hong Bang, Ho Chi Minh, Vietnam; 6 School of Medicine, Vietnam National University, Ho Chi Minh City, Ho Chi Minh, Vietnam; 7 Evidence Based Medicine Research Group & Faculty of Applied Sciences, Ton Duc Thang University, Ho Chi Minh City, Vietnam; 8 Department of Clinical Product Development, Institute of Tropical Medicine (NEKKEN), Nagasaki University, Sakamoto, Nagasaki, Japan

## Abstract

Glucose-6-Phosphate Dehydrogenase (G6PD) deficiency overlaps with malaria endemicity although it predisposes carriers to hemolysis. This fact supports the protection hypothesis against malaria. The aim of this systematic review is to assess the presence and the extent of protective association between G6PD deficiency and malaria. Thirteen databases were searched for papers reporting any G6PD alteration in malaria patients. Twenty-eight of the included 30 studies were eligible for the meta-analysis. Results showed absence of negative association between G6PD deficiency and uncomplicated falciparum malaria (odds ratio (OR), 0.77; 95% confidence interval (CI), 0.59–1.02; *p* = 0.07). However, this negative association happened in Africa (OR, 0.59; 95% CI, 0.40–0.86; *p* = 0.007) but not in Asia (OR, 1.24; 95% CI, 0.96–1.61; *p* = 0.10), and in the heterozygotes (OR, 0.70; 95% CI, 0.57–0.87; *p* = 0.001) but not the homo/hemizygous (OR, 0.70; 95% CI, 0.46–1.07; *p* = 0.10). There was no association between G6PD deficiency and total severe malaria (OR, 0.82; 95% CI, 0.61–1.11; *p* = 0.20). Similarly, there was no association with other malaria species. G6PD deficiency can potentially protect against uncomplicated malaria in African countries, but not severe malaria. Interestingly, this protection was mainly in heterozygous, being x-linked thus related to gender.

Glucose-6-phosphate dehydrogenase (G6PD) deficiency is an incomplete hereditary X-linked hemolytic disease. This enzymopathy is widespread in the tropics and subtropics, synchronizing with endemic or formerly endemic malaria^1^; which suggests that G6PD deficiency may have arisen, spread, or maintained in frequency through natural selection by malaria^2^,^3^. Although it is hard to detect G6PD deficient patients as most affected people are asymptomatic until they are exposed to triggers^4^, more than 400 million people are thought to be G6PD deficient^2^.

G6PD catalyzes the reaction in the pentose phosphate pathway that generates reduced form of NADPH, which is in turn responsible for glutathione (GSH) homeostasis^5^. GSH is an antioxidant, and together, these processes make cells more able to resist and control oxidative stress^2^. Inability of the erythrocytes to maintain GSH homeostasis results in oxidative stress and affects the integrity of the RBCs, giving rise to hemolysis. Optimum RBC redox status is required by malaria parasites for their survival, replication, and development^6^. This factor is diminished in G6PD deficient RBCs, supporting the protection hypothesis.

Although circumstantial evidence accumulated to support the hypothesis that G6PD deficiency is protective against severe fatal malaria^1^,^7^,^8^,^9^; there have been several arguments for^7^,^10^,^11^,^12^ and against^13^,^14^,^15^. Some researchers also argued that perhaps malaria may not be the only factor affecting the deficiency gene locus^16^. It remains to be clarified whether a direct association exists between G6PD deficiency and protection from malaria^11^,^17^. This systematic review and meta-analysis was undertaken to integrate all the informative studies focusing on the association between malaria and genetically determined G6PD deficiency to assess the presence and the extent of this association.

## Results

### Study selection

**Figure 1:**
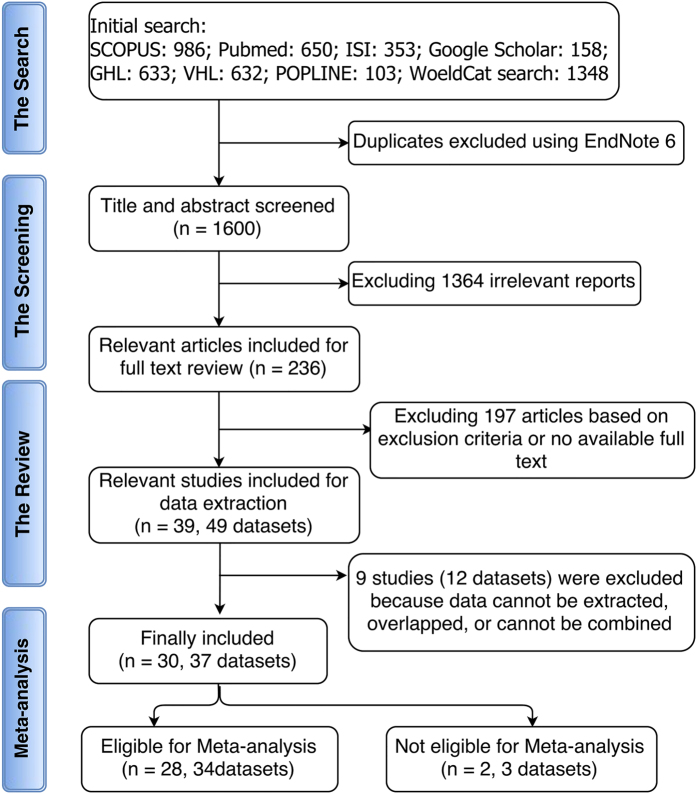
Flow diagram showing the method for the search, abstract screening, systematic review, and meta-analysis. The database search using the search strategy was cleaned up to exclude duplicates. Titles and abstracts were initially screened to include all studies (published before January 2014) describing any association between G6PD deficiency and malaria. In addition to not meeting the inclusion criteria, reviews, case studies, editor correspondences, and studies whose data could not be retrieved were excluded during the full-text review.

The initial search on public databases yielded 1600 study reports after excluding duplicates. Titles and abstracts screening resulted in exclusion of 1364 studies based on the exclusion criteria. Only 236 studies were considered eligible for inclusion for full-text review. Further 197 studies were excluded after full-text reading. A total of 39 studies were included, which comprises 49 data sets due to multi-center study reports. However, nine studies whose data either cannot be extracted, overlapped, or combined with the rest of the data, and thus were excluded. Among the 30 included studies, the species of *Plasmodium* are only *Plasmodium falciparum* in 17 studies^3^,^5^,^7^,^10^,^11^,^18^,^19^,^20^,^21^,^22^,^23^,^24^,^25^,^26^,^27^,^28^,^29^, only *Plasmodium vivax* in one study^30^, and combined species (*P. falciparum* with *P. vivax, P. falciparum* with *P. malariae, P. vivax* with *P. malariae*, and *P. falciparum* with *P. vivax* and *P. malariae*) in eight studies^14^,^15^,^31^,^32^,^33^,^34^,^35^,^36^. Jalloh 2004^32^, Tantular 1999^36^, and Kruatrachue 1962^15^ reported separate data for *P. vivax* which meta-analyzed with that of Leslie 2010^30^. The species cannot be determined in four studies^37^,^38^,^39^,^40^. Finally, a total of 28 studies containing 34 datasets were included in the quantitative data synthesis and meta-analysis (Fig. 1).

### Characteristics of included studies

The characteristics of the 30 included studies are described in details in Supplementary Table S1. The extracted items are described in the methodology section. Seventeen (17) studies with 21 datasets were on patients in Africa, 11 studies containing 14 datasets were performed in Asia while one study each was reported from Brazil and Papua New Guinea. All included studies used prospective method for data collection. Seven (7) studies were case-control studies; one was randomized double-blinded clinical trial while the rest were cross-sectional studies. Sixteen (16) studies were performed on children and infants only, while the other studies included children and adults. Only three studies used random sampling, the other adopted consecutive method for patient recruitment.

### Association of G6PD deficiency with protection from uncomplicated *P. falciparum* malaria

**Figure 2:**
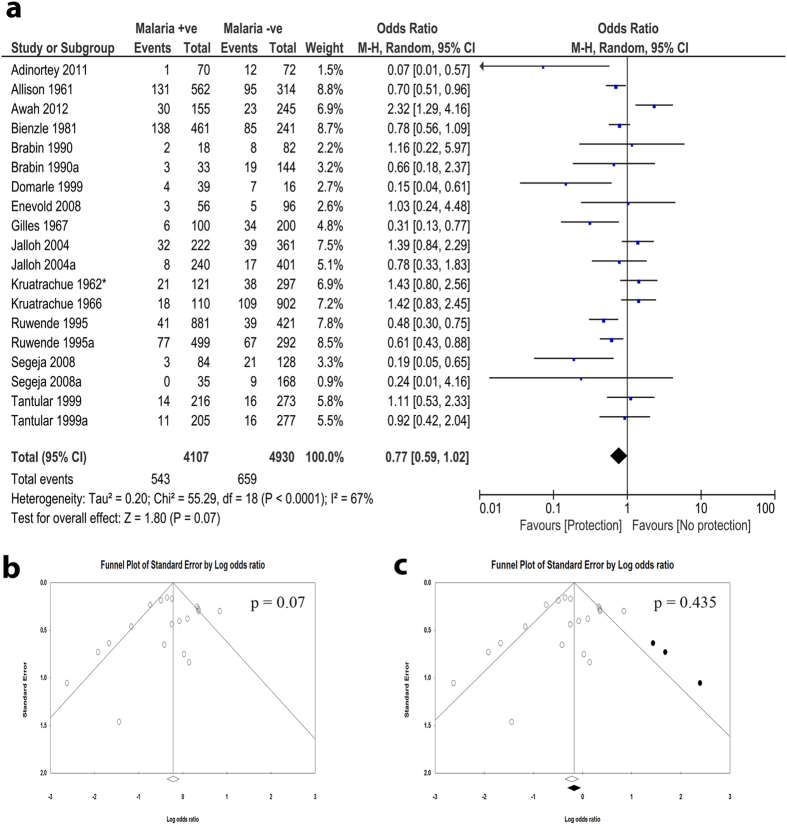
Association of G6PD deficiency with protection from uncomplicated falciparum malaria. (**a**) The forest plot of meta-analysis assessing the association between G6PD deficiency and prevalence of *P. falciparum* malaria. The comparators were malaria negative individuals. The observed results revealed absence of negative association between G6PD deficiency and *P. falciparum* malaria prevalence. **(b)** Funnel plot revealed the presence of publication bias towards negative association. **(c)** Trim and fill analysis led to further tilting away from the negative association, confirming the presence of publication bias among the included studies.

A meta-analysis was performed to assess the frequencies of G6PD deficiency between uncomplicated *P. falciparum* malaria and malaria negative individuals. The odds ratios (OR) from 19 datasets obtained from 14 studies^3^,^5^,^7^,^10^,^14^,^15^,^18^,^20^,^22^,^24^,^29^,^31^,^32^,^36^ were pooled for this meta-analysis. There was significant heterogeneity among the studies; thus, random effect model was applied for the meta-analysis. The combined OR revealed absence of negative association between G6PD deficiency and uncomplicated malaria (OR, 0.77; 95% confidence interval (CI), 0.59–1.02; n, 19; *p* = 0.07) (Fig. 2a). There was publication bias in favor of studies with negative association (Fig. 2b). When the cut and fill method as proposed by Duval *et al*.^41^ was applied, the condition negative association was further lost (OR, 0.88; 95% CI, 0.66–1.17; n, 22; *p* = 0.435).

Sensitivity analysis was performed to investigate the effect of a single study on the outcome measures. We observed significant effects of single studies on the meta-analysis result. Interestingly, the exclusion of any of four studies^10^,^14^,^15^,^32^ led to appearance of significant negative association in the meta-analysis (Supplementary Table S2). The cumulative meta-analysis also showed that the inclusion of single studies, especially four studies^10^,^14^,^18^,^31^, affected the outcome effect measures (Supplementary Table S3).

### Subgroup analysis shows that protection from uncomplicated *P. falciparum* malaria is significantly observed in heterozygotes

**Figure 3:**
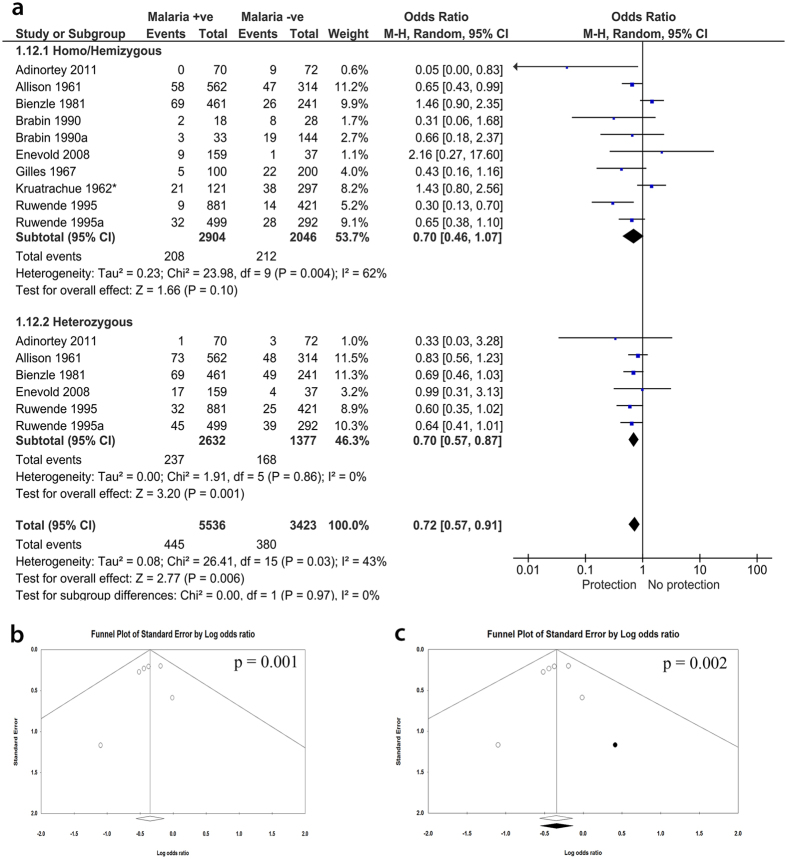
Meta-analysis forest plot for the association of G6PD with falciparum malaria. (Subgroup analysis by genotypes): homo/hemi or heterozygous). (**a**) Present the meta-analysis forest plot of the association of G6PD deficiency and falciparum malaria, grouped by genotypes. The heterozygous females were compared to a combined group comprising hemizygous males and homozygous female. There was a strong negative association between G6PD deficiency and malaria for the heterozygous. (**b**) Funnel plot of the heterozygous subgroup represents presence of publication bias towards negative association. (**c**) Trim and fill analysis indicate that the publication bias is too minimal to affect the significant negative association.

Subgroup analysis was performed to identify the effect of population subgroups on the meta-analysis. We grouped data into genotypes by separately analyzing studies that reported different genotypes. When homo/hemizygous or heterozygous were analyzed separately, the outcome effect measures were significant for heterozygous (OR, 0.70; 95% CI, 0.57–0.87; n, 6; *p* = 0.001), but not for homo/hemizygous (OR, 0.70; 95% CI, 0.46–1.07; n, 10; *P* = 0.10) indicating that the association is only present in heterozygous subgroup (Fig. 3a). However, there was a publication bias observed by funnel plot in favor of the protection (Fig. 3b). Trim and fill methods of Duval indicated that this publication bias is so minimal to affect the significant association in the heterozygous subgroup (OR, 0.71; 95% CI, 0.57–0.88; n, 7; *P* = 0.002) (Fig. 3c). Also, sensitivity analysis showed there was no single study effect on this association (Supplementary Table S4). Subgroup analysis by age revealed negative association with children only (OR, 0.72; 95% CI, 0.53–0.98; n, 9; *P* = 0.04) but not with children and adult subgroup (Supplementary Fig. S1). When the genders were analyzed separately, the significant association was only present with female subgroup (OR, 0.70; 95% CI, 0.50–0.98; n, 7; *P* = 0.04) (Supplementary Fig. S2). This corroborates the significant association for heterozygous subgroup since G6PD enzymopathy is sex-linked.

### Subgroup analysis by the location of the participants

**Figure 4:**
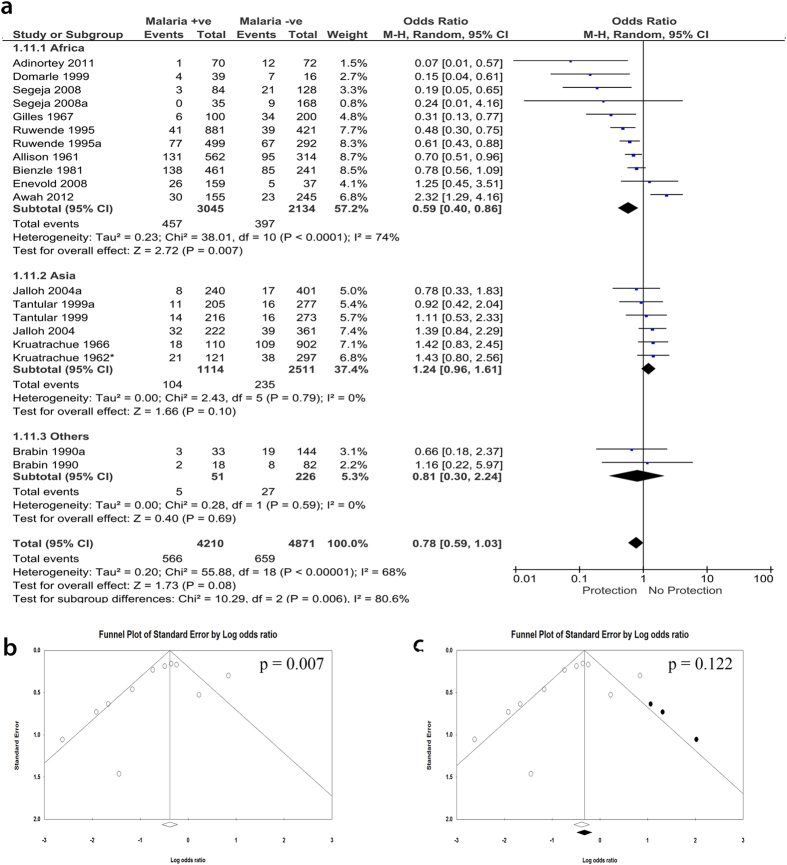
Meta-analysis forest plot for the association of G6PD with falciparum malaria. (Subgroup analysis by continents where each study was performed (Africa, Asia, or others (Papua New Guinea)). (**a**) The forest plot of the subgroup analysis showing an association of G6PD deficiency and falciparum malaria, sub-grouped into the continents where study site is located. A statistically significant negative association was observed from the meta-analysis of studies on African subjects. No association was observed for studies performed in Asia. Also, the two data sets of a study from Papua New Guinea showed no association. (**b**) Funnel plot of studies performed in African. (**c)** Trim and fill analysis showing presence of publication bias in studies performed in Africa.

Interestingly, when the studies were grouped according to the continents where the studies were performed, we observed that the negative association between G6PD and malaria was only observed for studies performed in Africa (OR, 0.59; 95% CI, 0.40–0.86; n, 11; *P* = 0.007) (Fig. 4a). The studies performed in Asia showed no significant association (OR, 1.24; 95% CI, 0.96–1.61; n, 6; *P* = 0.1) (Fig. 4a). However, although sensitivity analysis did not show any single study or small study effect on the observed negative association, funnel plot showed evidence of publication bias in favor of studies with negative association. When the cut and fill method as proposed by Duval *et al*.^41^ was applied, the statistical significance of the association was lost (OR, 0.74; 95% CI, 0.49–1.10; n, 14; *P* = 0.122) (Fig. 4b and c).

### Association of G6PD deficiency with severe falciparum malaria

Having identified association between G6PD deficiency and mild *P. falciparum* malaria, we assessed whether G6PD deficiency is also associated with protection from severe *P. falciparum* malaria. For this analysis, the comparator was uncomplicated falciparum malaria cases. Different severe falciparum malaria symptoms were considered together and then separately in relation to uncomplicated malaria. There was no association between G6PD deficiency and severe falciparum malaria, in relation to uncomplicated malaria (OR, 0.82, 95% CI, 0.61 to 1.11; n, 114; *P* = 0.20) (Supplementary Fig. S3). Even when the types of severe falciparum malaria were considered separately, we observed significant negative association between G6PD deficiency and hyperparasitemia (OR, 0.73; 95% CI, 0.56–0.95; n, 8; *P* = 0.02), but not between G6PD deficiency and severe anemia or cerebral malaria (Supplementary Fig. S3). When doing sensitivity analysis to investigate the effect of one study removal on the observed negative association between G6PD and hyperparasitemia, there was a significant effect of single studies on the observed results. Exclusion of one of two studies, Gilles 1967^24^ and Allison 1961^7^, led to loss of the observed significant association (Supplementary Table S5). The analysis in this section did not show evidence of publication bias as evident in the good symmetry of the funnel plots, except for the hyper-parasitemia, with which, we observed a minimal publication bias that did not affect the results (Supplementary Fig. S3).

### G6PD deficiency was not associated with malaria due to other species of *Plasmodium*

**Figure 5:**
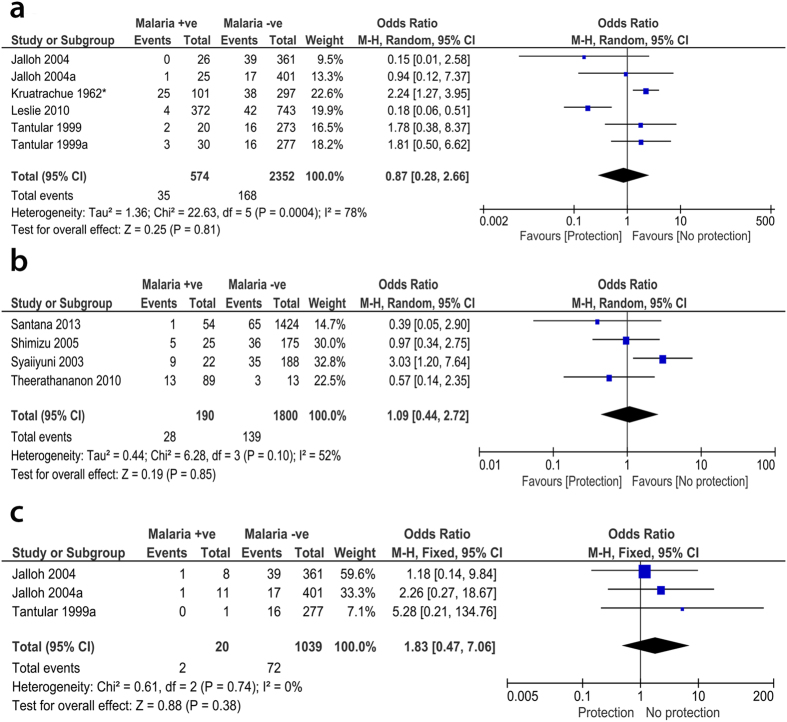
Meta-analysis forest plot for the association of G6PD with other species apart from *P. falciparum* malaria. There was no negative association between G6PD and *P. vivax* (**a**), *P. malariae* (**c**), or even nondetermined species (**b**).

We also investigated the association of G6PD with other species of *Plasmodium* including *P. vivax, P. malariae* or combined co-infections. For *P. vivax* malaria, the OR from six datasets obtained from four papers^15^,^30^,^32^,^36^ showed that there was no association between G6PD and vivax malaria (OR, 0.87; 95% CI, 0.28–2.66; n, 6; *P* = 0.81), however, the two non-analyzable papers, Louicharoen 2009^34^ and Khim 2013^33^, reported significant association of G6PD with *P. vivax*. Similarly, there was no negative association between G6PD and malaria in patients infected with *P. malariae,* as reported by three datasets from two papers^32^,^36^ (OR, 1.83; 95% CI, 0.47–7.06; n, 3; *P* = 0.38) (Fig. 5a and c).

When combined co-infection with both *P. falciparum* and *P. vivax* was considered, the OR from five datasets derived from three papers^14^,^32^,^36^ also showed no negative association (OR, 0.95; 95% CI, 0.50–1.80; n, 5; *P* = 0.87). Besides the previous co-infection, the OR from five datasets of three papers^32^,^35^,^36^ also indicated no association with co-infection by *P. falciparum* and *P. malariae* (OR, 1.56; 95% CI, 0.29–8.34; n, 5; *P* = 0.60). Similarly, the pooled OR from four datasets reported in Jalloh 2004^32^ and Tantular 1999^36^ showed no association when combined *P. vivax* and *P. malariae* (OR, 1.17; 95% CI, 0.22–6.35; n, 3; *P* = 0.85) or combined *P. falciparum, P. vivax,* and *P. malariae* (OR, 1.90; 95% CI, 0.42–8.51; n, 4; *P* = 0.40) co-infections were considered (Supplementary Fig. S4). We could not determine the species of *Plasmodium* reported in four analyzable studies^37^,^38^,^39^,^40^. Nevertheless, the pooled OR from these studies did not show significant association (OR, 1.09; 95% CI, 0.44–2.72; n, 2; *P* = 0.85) (Fig. 5b).

## Discussion

We observed absence of negative association between G6PD deficiency not only with uncomplicated falciparum malaria but also with severe malaria (Fig. 2). However, single studies may have dramatically affected this analysis and inference. For instance, the exclusion of any of four studies^10^,^14^,^15^,^32^ led to appearance of negative association in the meta-analysis. There was also evidence of publication bias in favor of negative association (protection). Only one study by Adinortey *et al*.^5^ was published in a non-peer-reviewed journal among the included studies, however, it does not affect the meta-analysis as shown in the sensitivity analysis. Similarly, there was no significant association between G6PD deficiency and other species of malaria, including *P. vivax* and *P. malariae* or combinations of any two or all the three species. Due to the insufficient number of reports on these species, we could not make any evident conclusion on the exact relationship especially after considering the results of the two non-analyzable papers. Therefore, more studies investigating the association between these species and G6PD deficiency are highly recommended. A negative association was found in studies performed in Africa as compared to Asia and other continents, albeit with presence of publication bias. These results may provide evidence in support of the G6PD-malaria protection hypothesis in Africa. G6PD deficiency is endemic in Africa and the African G6PD deficient patients have relatively higher enzyme activity and milder consequences than Mediterranean or Asian patients^3^,^42^. This may be one possible explanation the observed G6PD associated protect from falciparum malaria in Africa. The population history, archeology, and human genetics indicate that the age of this selection by malaria is more than 5,000 years old in Africa^3^,^43^. Moreover, the frequencies of deficiency alleles in most G6PD deficient populations in endemic areas do not exceed 40%^3^,^44^. Interestingly, the pooled data from Asian countries show no significant association. There may be a confounding relationship between location and the observed negative association in the children subgroup because of the skewing effect of African studies in favor of negative association (Supplementary Fig. S1)^14^,^15^,^39^,^40^. This suggests that other than endemicity, ethnicity could modify the effect of G6PD deficiency in the study. While the female homozygous and male hemizygous genotypes showed no association with uncomplicated malaria protection, the female heterozygous genotype showed highly significant association with protection from malaria. Unlike in homo/hemizygous subgroup, the association observed for heterozygous subgroup had no indication of heterogeneity or publication bias. This association with heterozygous females is highly supported by the study of Uyoga *et al*.^45^ which also indicated that hemizygous males are not only unprotected from malaria, but may be at high risk of sever malaria. This is due to the fact that G6PD-deficient red blood cells are prone to early destruction by oxygen free radicals^46^. Usanga and Luzzatto hypothesized a prestigious mechanism that explains the association with heterozygous females^47^. They observed that the parasite undergoes habitual changes when passing through successive G6PD-deficient red cells in order to be more adaptive. These changes resulted in production of the parasite’s own enzyme which in turn resulted in survivability and replication of the parasite^48^. As the heterozygous females are genetically mosaic, the parasite failed to survive^47^. Supportingly, when subgrouping the patients according to the enzymatic activity, our analysis revealed that the activity of the enzyme has no effect on the association, although only two analyzable papers reported the enzymatic activity for *P. falciparum*.

Studies have demonstrated the presence of G6PD variants in geographically diverse locations, endemic or formerly endemic for malaria; suggesting the likelihood of selection by malaria^2^. However, if malaria were the sole selecting factor on the G6PD gene with no other deleterious factor, an increase in the proportion of the genotype conferring G6PD enzymopathy would be expected to replace the normal allele^1^,^49^. The absence of clear evidence of this selection supports the hypothesis that the net protection observed is dependent on the balance between the malaria selection and other deleterious effects of G6PD enzymopathy^16^. It further confirms that there may be more than one selection factors on this gene, with the effect of the advantageous factors (e.g. malaria) countering the effects of factors that confer net disadvantage (e.g. hemolysis)^7^,^8^,^16^. As a matter of fact, homozygous females and hemizygous males are more severely affected by the hemolytic effects of G6PD deficiency^45^,^49^. This may, therefore, explain the better protection from malaria in the heterozygous females.

Conversely, G6PD deficiency was not associated with protection from severe falciparum malaria. Severe malaria is characterized by severe anemia, hyperparasitemia, and cerebral malaria. The net negative odds ratios observed for severe anemia and cerebral malaria were not statistically significant. Peculiarly, the hyperparasitemia showed significant negative association with G6PD deficiency in relation to low parasitemia. There was minimal publication bias in favor of protection that did not affect the significant association. Association with hyperparasitemia may however not only indicate severity of infection but also reinfection rate. These severity factors are associated with or arguably exacerbated by hemolysis, a characteristic feature of G6PD deficiency. However, the hemolytic effect of G6PD deficiency is self-limiting, affecting mainly older erythrocytes than the newly formed ones; thereby establishing a balance between the factors. Thus, the concept of protection from severe malaria or absence thereof remains conflicting^10^,^17^,^50^.

The inherent disadvantage of G6PD deficiency due to the associated hemolysis could be one of the factors that account for the absence of consensus on the G6PD-malaria protection hypothesis. Also, the presence of other factors that could protect from malaria, especially hemoglobinopathies like sickle cell anemia (HbS) which also overlaps with G6PD and malaria endemicity, is considered one of the major confounders of our analysis and limitations of this study.

To recapitulate, we inferred from this analysis that G6PD deficiency may not generally protect against uncomplicated falciparum malaria, *P. vivax,* or *P. malariae*. However, the analysis was sensitive to the effect of individual studies exclusion of which resulted in negative association with uncomplicated falciparum malaria, albeit with evidence of publication bias in favor of malaria protection. The protection was found only in African countries but not in Asian. Comparing the genotypes, the negative association was found only in heterozygous females, but not in a combined group comprising homozygous females and hemizygous males. Similarly, the negative association was present in studies including only children but not with combined group comprising children and adults. With the exception of hyperparasitemia, G6PD deficiency showed no association with severe malaria. Further studies are needed to investigate the mechanism of association with heterozygous females.

## Methods

### Registration of study protocol

This study was performed according to the recommendations of the PRISMA statement^51^ during all steps of the study. The protocol for this study was prepared prior to the start of the study and was registered in PROSPERO with identification number CRD42014007282.

### Study eligibility criteria

Only original articles that report the association of any G6PD alteration in enzymatic activity and genetic variant and malaria were included. There was no restriction made with respect to language, publication year and study design. Case reports, reviews, theses, conference proceedings were excluded. Two reviewers independently extracted the data and subsequently verified by two other reviewers. Any disagreements were resolved by discussion and consensus between the reviewers and a third party, if necessary.

### Search strategy

Thirteen electronic databases were searched including; PubMed, Scopus, Google Scholar, ISI, WHO Global Health Library (GHL), Cochrane Review Library, IBECS, POPline, Virtual Health Library (VHL), WorldCat, New York Academy of Medicine Grey Literature Report, System for Information on Grey Literature in Europe (SIGLE), and African Journals Online databases. Searches were conducted to find relevant studies published before January 2014. The initial search strings were done using PubMed and Scopus databases using the broad search term: “((Glucose-6-phosphate dehydrogenase OR G6PD) AND (Malaria[Mesh] OR Antimalarial[Mesh]) and (INDEXTERMS (glucose-6-phosphate dehydrogenase OR G6PD) AND (malaria OR antimalarial))”. Subsequently, this search string was modified to contain search terms suitable for each database. The details of search strategy and terms used in each database are presented in Supplementary File S1.

### Study inclusion criteria and study selection

All original studies (published before January 2014) describing association between G6PD deficiency and malaria were included in this systematic review. All eligible studies irrespective of publication type, study design, language and publication date were considered in the qualitative systematic review. There was no restriction on certain population, age, race, ethnicity, or geographic area. Only variables reported by two or more studies and whose primary data can be extracted were included in the meta-analysis. We limited included studies to those performed on human subjects. Reviews, case studies, case series, editor correspondences, news, letters, book chapters and studies whose data could not be reliably retrieved or extracted were excluded. We omitted studies whose data is overlapped with another included study. Two reviewers independently performed initial screening and study selection. Preliminary assessment of the title and abstracts was performed to identify relevant articles. Thereafter, full texts of eligible articles were downloaded and reviewed for qualitative analysis and potential inclusion in the data synthesis. Inclusion of a study by both reviewers was conclusive while discrepancies and disagreements as regards study eligibility were resolved by discussion and/or consensus with a third reviewer.

### Data extraction

The data extracted from the included reports include: the first author, year of publication year of data, study design, data collection (prospective or retrospective), country and city of origin, characteristics of participant population (gender, age, and the type of malaria), method for genetic variants detection, number of included individuals (case and control groups), outcomes and times of evaluation. When there was no data available or if there were obvious errors (such as typographical errors, incorrect calculation, or incorrect factors designations) in the original publication, two successive emails were sent to corresponding authors for clarification. When no clarification or genotype information was obtained after at least two emails, we will report such studies as “no data available”. Identification and elimination of potentially overlapped datasets were done under extensive care, especially for multiple reports by the same group reporting the same factors on the same subjects.

### Meta-analyses

Meta-analysis to pool data from eligible studies was performed on both RevMan v5.2^52^ and Comprehensive Meta-Analysis v2 software. 2 × 2 contingency tables were generated and the odds ratio with the corresponding 95% CI were calculated for dichotomous outcomes. For outcomes with continuous variables, the input data were mean and standard deviation (SD) with the standardized mean difference (SMD) as the effect measure. When standard deviation was not available, it was computed with the calculator function in RevMan v5.2 by inputting other supplied data (e.g. mean, standard error of mean, *p*-value etc.), if available. Forest plots showing the respective OR or SMD with their corresponding 95% CI for each dataset and for the pooled data were generated. The overall effect was measured by Z-statistics with statistical significance set at *p* < 0.05. More clarifications and in-depth analysis were achieved through subgroup analysis.

### Test of heterogeneity between studies

The Cochrane Q (*Chi*^2^ test) and *I*^*2*^ statistics^53^ were used to assess inconsistency among studies. For *Chi*^2^ test of heterogeneity, statistical significance was set as *p* < 0.10. Fixed-effects model was adopted when there was a lack of significant heterogeneity (in *Chi*^2^*, p* > 0.10 or *I*^*2*^ values < 50%), otherwise heterogeneity among studies is assumed and random-effects model with weighting of the studies was applied^54^.

### Sensitivity analysis and assessment of publication bias among studies

For sensitivity analysis, each individual study in each meta-analysis was sequentially excluded in the analysis to examine the effect of a single study on the outcome of the meta-analysis. Begg’s funnel plots were used to initially assess publication bias across the reviewed studies^55^,^56^. The funnel plots were created by plotting the OR against the standard error of the logarithm of OR. When there was a publication bias, cut and fill method as proposed by Duval and Tweedie^41^ was conducted to indicate the effect of this publication bias on the estimated effect size.

## Additional Information

**How to cite this article**: Chibunna Mbanefo, E. *et al*. Association of glucose-6-phosphate dehydrogenase deficiency and malaria: a systematic review and meta-analysis. *Sci. Rep.* **7**, 45963; doi: 10.1038/srep45963 (2017).

**Publisher's note:** Springer Nature remains neutral with regard to jurisdictional claims in published maps and institutional affiliations.

## Supplementary Material

Supplementary Information
