# Supplementary material for: Association of glucose-6-phosphate dehydrogenase deficiency and malaria: a systematic review and meta-analysis
Source: Sci Rep. 2017 Apr 6;7:45963. doi: 10.1038/srep45963 (PMC5382680; doi:10.1038/srep45963)
Supplement: Supplementary Information [file srep45963-s1.pdf]

Association of glucose-6-phosphate dehydrogenase deficiency and malaria: a systematic review and meta-analysis

Evaristus Chibunna Mbanefo[1,2,*], Ali Mahmoud Ahmed[3,*], Afaf Titouna[1,*], Ahmed Elmaraezy[3,*], Nguyen Thi Huyen Trang[4], Nguyen Phuoc Long[5], Nguyen Hoang Anh[6], Tran Diem Nghi[6], Bui The Hung[5], Mai Van Hieu[6], Nguyen Ky Anh[5], Nguyen Tien Huy[7,8], & Kenji Hirayama[1]

[1]Department of Immunogenetics, Institute of Tropical Medicine (NEKKEN), Nagasaki University, Sakamoto, Nagasaki, Japan.
[2]Department of Parasitology and Entomology, Nnamdi Azikiwe University, P.M.B. Awka, Nigeria.
[3]Faculty of Medicine, Al-Azhar University, Cairo, Egypt.
[4]University of Medicine and Pharmacy at Hue City, Hue City, Vietnam.
[5]University of Medicine and Pharmacy at Ho Chi Minh City, Hong Bang, Ho Chi Minh, Vietnam.
[6]School of Medicine, Vietnam National University, Ho Chi Minh City, Ho Chi Minh, Vietnam.
[7]Evidence Based Medicine Research Group & Faculty of Applied Sciences, Ton Duc Thang University, Ho Chi Minh City, Vietnam.
[8]Department of Clinical Product Development, Institute of Tropical Medicine (NEKKEN), Nagasaki University, Sakamoto, Nagasaki, Japan.

[*]These authors contributed equally to this study

Correspondence and requests for materials should be addressed to N.T.H. (email: nguyentienhuy@tdt.edu.vn) or K.H. (email: hiraken@nagasaki-u.ac.jp)

File S1. Search strategy

Nine electronic searches were conducted to find relevant studies. Initial searches were done using PubMed and Scopus databases using the broad search term: “((Glucose-6-phosphate dehydrogenase OR G6PD) AND (Malaria[Mesh] OR Antimalarials[Mesh]) and (INDEXTERMS (glucose-6-phosphate dehydrogenase OR g6pd) AND (malaria OR antimalarials))”. We removed reviews, conference abstracts and editorials. For the search on ISI database, we used the term: “((glucose-6-phosphate dehydrogenase OR g6pd) AND (malaria OR antimalarials))”, but this search was refined by: [excluding] document type as :(Meeting abstract or note or book chapter or review or editorial material or news item). For Google Scholar, first, we performed the search with the exact phrase: glucose 6 phosphate dehydrogenase, with at least one of the words: malaria/antimalarial, further using (allintitle: malaria OR antimalarial "glucose 6 phosphate dehydrogenase") and then with (allintitle: malaria OR antimalarial "G6PD"). With WHO Global Health Library search, the used search term was: ((glucose 6 phosphate dehydrogenase) OR (G6PD)) and (Malaria OR Antimalarials). For Cochrane Library, IBECS, we filled the term: ((glucose 6 phosphate dehydrogenase) OR (G6PD)) and (Malaria OR Antimalarials)). POPLINE advanced search was performed using the term: (All Fileds: glucose 6 phosphate dehydrogenase OR g6pd AND keywords: malaria or antimalarials). While the search on New York Academy of Medicine Grey Literature Report and SIGLE (System for Information on Grey Literature in Europe) databases were searched with broad term: ((glucose 6 phosphate dehydrogenase) OR (G6PD)) and (Malaria OR Antimalarials).

Table S1. Characteristics of included studies

ID	Country	Year of recruitment	Study design	Subject of study	Total sample size.	Age		Gender (Female)		Test detection of malaria	Malaria strain	PCR related methods for G6PD		Phenotype method for G6PD (enzyme activity)	**Malaria patients**	
						Median/Max **	Range/SD*	N	%			PCR type	Other tests		**N**	**%**
**Fanello 2008**	Rwanda	2005-2006	RCT	Infant and children	792	NA	0.5-4.9y	NA	NA	NA	Falciparum	PCR-RFLP	NA	NA	702	**89**
**Guindo 2007**	Mali	1997-1998, 2001-2004	case control	children	3197	NA	NA	1640	51.3	NA	Falciparum	PCR-RFLP	NA	NA	2765	**86**
**Guindo 2007a**	Mali	1997-1998, 2001-2004	case control	children	3197	NA	NA	1640	51.3	NA	Falciparum	PCR-RFLP	NA	NA	432	**14**
**Adinortey 2011**	Ghana	NA	case control	NA	142	NA	NA	65	45.8	Giemsa staining method	Falciparum	NA	NA	MetHb reduction test	70	**49**
**Allison 1961**	Tanganyika	NA	case control	Infant, children	876	NA	0.33-4y	452	51.6	Blood film	Falciparum	NA	NADP	NA	562	**64**
**Awah 2012**	Nigeria	NA	cross section	Children and adult	400	NA	5-50y	156	39.0	Giemsa or Leishman stain	Falciparum	NA	NA	Methaemoglobin	155	**39**
**Awah 2008**	Cameroon	NA	Cross section	children and adult	80	NA	3-70y	NA	NA	Giemsa or Leishman stained	Falciparum	NA	NA	flurorescent screening	80	**100**
**Bienzle 1979**	Nigeria	NA	Cross setion	children	702	NA	1-6y	323	46.0	NA	Falciparum	NA	NA	gel electrophoresis	461	**66**
**Bienzle 1980**	Nigeria	1969-1970	cross section	children	702	NA	0.75-6y	323	46.0	Blood film	Falciparum	NA	NA	NA	461	**66**
**Brabin 1990**	Papua New Guinea	1987-1988	Cross section	NA	302	NA	NA	0	0.0	NA	Falciparum, another species	NA	NA	flurorescent screening	285	**94**
**Domarle 1990**	Gabon	1998	Cross sectional	Children	61	10.08y	4.99y*	27	44.3	Blood smear	Falciparum	NA	NADPH	NA	41	**67**
**Dunyo 2011**	The Gambia	2004	RCT	Infant + children	618	5y	0.5-11y	337	54.5	Blood film	Falciparum	PCR-RFLP	NA	NA	618	**100**
**Dunyo 2011a**	The Gambia	2004	RCT	Infant + children	620	4.8y	0.5-10.8y	291	46.9	Blood film	Falciparum	PCR-RFLP	NA	NA	620	**100**
**Gilles 1967**	Nigeria	NA	cross section	children	300	NA	0.5-4y	143	47.7	NA	Falciparum	NA	NA	NA	100	**33**
**Enevold 2008**	Tanzania	2001	Case control	Infant, children, adult	196	5.9y	5.2-6.8y	106	54.1	Blood slide	Falciparum	PCR - SSOP	NA	NA	159	**81**
**Jalloh 2004**	Myanmar	2002-2003	cross section	children and adults	650	21.5y	1-75 yrs	309	47.5	thin aNA thick smears	Falciparum, Vivax, Malariae	NA	NADPH	NA	289	**44**
**Jalloh 2004a**	Indonesia	2002-2003	cross section	children and adults	709	NA	NA	335	47.2	thin aNA thick smears	Falciparum, Vivax, Malariae	NA	NADPH	NA	308	**43**
**Khim 2013**	Cambodia	2010-2012	Cross sectional	Children + adult	965	26.5y	25.8-27.2y	226	23.4	Blood film	Falciparum, Vivax	PCR-RFLP	NA	NA	965	**100**
**Kruatrachue 1966**	Thailand	NA	cross sectional	infant, children	1012	NA	0-3y	483	47.7	Blood film	Falciparum, vivax	NA	NA	NA	110	**11**
**Kruatrachue 1962**	Thailand	NA	cross sectional	children	519	NA	1-9y	0	0.0	Blood film	Falciparum, vivax	NA	NA	NA	212	**41**
**Lell 1998**	Gabon	1995-1996	case-control	children	200	3.7y	NA	122	61.0	NA	Falciparum	PCR	subsequent hybridization	NA	200	**100**
**Leslie 2010**	Pakistan	2006	case control	children	1115	13.6y	NA	641	57.5	NA	Vivax	PCR	NA	Colorimetry	372	**33**
**Louicharoen 2009**	Thailand	1998-2005	cross section	children and adults	227	NA	NA	92	40.5	NA	Vivax	NA	LRH test	NA	227	**100**
**Louicharoen 2009a**	Thailand	1998-2005	cross section	children and adults	400	NA	NA	175	43.8	NA	Falciparum	NA	LRH test	NA	400	**100**
**Nkuo-Akenji 2004**	Cameroon	2002	cross sectional	Children and Adult	222	NA	1-60y	113	50.9	Blood film	Falciparum, malariae	NA	Met-haemoglobin reduction test	NA	150	**68**
**Myint-Oo 1995**	Myanmar	NA	Cross sectional	Adult	383	NA	19-45y	0	0.0	Thick aNA thin blood smear	Falciparum	NA	Methaemoglobin reduction test	NA	383	**100**
**Orimadegun 2011**	Nigeria	NA	Cross sectional	Infant, Children	930	2.9y	0.5-12y	472	50.8	Blood film	Falciparum	NA	NADPH	NA	930	**100**
**Ruwende 1995**	Gambia	NA	case control	children	1303	10y **	NA	603	46.3	NA	Falciparum	PCR-SSOP	NA	spectrophotometric methods	881	**68**
**Ruwende 1995a**	Kenya	NA	case control	children	804	10y **	NA	405	50.4	NA	Falciparum	PCR-SSOP	NA	spectrophotometric methods	506	**63**
**Santana 2013**	Brazil	2009 to 2010	cross sectional	Children and Adult	1478	NA	1-65y	0	0.0	Blood film/microscopy	NA	PCR-RFLP	NA	Enzymatic activity kit	54	**4**
**Segeja 2008**	Tanzania	2003	cross sectional	Children and Adult	212	NA	0.5-45y	123	58.0	Blood film/microscopy	Falciparum	NA	NA	NADPH Fluorescent test	84	**40**
**Segeja 2008a**	Tanzania	2003	cross sectional	Children and Adult	203	NA	0.5-45y	125	61.6	Blood film/microscopy	Falciparum	NA	NA	NADPH Fluorescent test	19	**17**
**Shimizu 2005**	Indonesia	NA	cross sectional	children	210	11.6y	6-17y	110	52.4	PCR	NA	NA	NA	Formazan ring method	25	**12**

**Syaiiyuni 2003**	Indonesia	1996 to 2002	cross sectional	children	210	NA	NA	11 7	55.7	Blood film/microscopy	NA	PCR-RFLP	NA	Formazan ring method	22	**11**
**Tantular 1999**	Indonesia	1997-1998	Cross sectional	NA	2205	NA	NA	10 93	49.6	Blood film, Antigen test (pLDH), Giemsa-stained blood smear	Falciparum, Vivax, and Malariae	NA	NA	Rapid G6PD test, Formazan ring test	273	**24**
**Tantular 1999a**	Myanmar	1997-1998	Cross sectional	NA	2205	NA	NA	10 93	49.6	Blood film, Antigen test (pLDH), Giemsa-stained blood smear	Falciparum, Vivax, and Malariae	NA	NA	Rapid G6PD test, Formazan ring test	277	**26**
**Theerathana non 2010**	Thailand	2006	Cross sectional	Children and Adult	102	40.0 1yr	15-75y	62	60.8	Blood film, Antigen test (pLDH), Giemsa-stained blood smear	NA	NA	NADPH	Fluorescence spot test	89	**87**

Table S3. Sensitivity Analysis (Effect of individual studies sorted by N)

		Effect of exclusion of one study				
**Studies**	**Order**	**OR**	**Lower CI**	**Higher CI**	**Z-score**	**p-value**
Adinortey 2011	1	0.81	0.62	1.06	1.56	0.12
Allison 1961	2	0.77	0.56	1.05	1.64	0.10
Awah 2012	3	0.73	0.56	0.94	2.41	0.02
Bienzle 1981	4	0.76	0.56	1.04	1.70	0.09
Brabin 1990	5	0.77	0.58	1.02	1.84	0.07
Brabin 1990a	6	0.78	0.58	1.03	1.73	0.08
Domarle 1999	7	0.82	0.62	1.07	1.48	0.14
Enevold 2008	8	0.77	0.58	1.02	1.83	0.07
Gilles 1967	9	0.81	0.62	1.08	1.45	0.15
Jalloh 2004	10	0.74	0.56	0.98	2.06	0.04
Jalloh 2004a	11	0.77	0.58	1.03	1.75	0.08
Kruatrachue 1962*	12	0.74	0.56	0.99	2.06	0.04
Kruatrachue 1966	13	0.74	0.56	0.98	2.07	0.04
Ruwende 1995	14	0.81	0.61	1.08	1.45	0.15
Ruwende 1995a	15	0.78	0.58	1.06	1.57	0.12
Segeja 2008	16	0.82	0.62	1.07	1.46	0.14
Segeja 2008a	17	0.78	0.59	1.03	1.72	0.08
Tantular 1999	18	0.76	0.56	1.01	1.90	0.06
Tantular 1999a	19	0.76	0.57	1.02	1.81	0.07
Over all		0.77	0.59	1.02	1.80	0.07

Table S4. Sensitivity Analysis by Cumulative Meta-analysis sorted by N

Studies	Order																		
Adinortey 2011	1	1	1	1	1	1	1	1	1	1	1	1	1	1	1	1	1	1	1
Allison 1961		2	2	2	2	2	2	2	2	2	2	2	2	2	2	2	2	2	2
Awah 2012			3	3	3	3	3	3	3	3	3	3	3	3	3	3	3	3	3
Bienzle 1981				4	4	4	4	4	4	4	4	4	4	4	4	4	4	4	4
Brabin 1990					5	5	5	5	5	5	5	5	5	5	5	5	5	5	5
Brabin 1990a						6	6	6	6	6	6	6	6	6	6	6	6	6	6
Domarle 1999							7	7	7	7	7	7	7	7	7	7	7	7	7
Enevold 2008								8	8	8	8	8	8	8	8	8	8	8	8
Gilles 1967									9	9	9	9	9	9	9	9	9	9	9
Jalloh 2004										10	10	10	10	10	10	10	10	10	10
Jalloh 2004a											11	11	11	11	11	11	11	11	11
Kruatrachue 1962												12	12	12	12	12	12	12	12
Kruatrachue 1966													13	13	13	13	13	13	13
Ruwende 1995														14	14	14	14	14	14
Ruwende 1995a															15	15	15	15	15
Segeja 2008																16	16	16	16
Segeja 2008a																	17	17	17
Tantular 1999																		18	18
Tantular 1999a																			19
	1	2	3	4	5	6	7	8	9	10	11	12	13	14	15	16	17	18	19
**OR**	**0.07**	**0.28**	**0.7**	**0.85**	**0.88**	**0.86**	**0.72**	**0.75**	**0.67**	**0.75**	**0.76**	**0.82**	**0.87**	**0.82**	**0.8**	**0.75**	**0.74**	**0.76**	**0.77**
**Lower CI**	**0.01**	**0.03**	**0.2**	**0.45**	**0.49**	**0.51**	**0.42**	**0.45**	**0.41**	**0.48**	**0.51**	**0.56**	**0.61**	**0.58**	**0.59**	**0.55**	**0.55**	**0.57**	**0.59**
**Higher CI**	**0.57**	**2.58**	**2.4**	**1.06**	**1.57**	**1.45**	**1.24**	**1.24**	**1.09**	**1.16**	**1.13**	**1.19**	**1.23**	**1.14**	**1.08**	**1.02**	**1.01**	**1.02**	**1.02**
**Z-score**	**-2.5**	**-1.1**	**-0.5**	**-0.5**	**-0.4**	**-0.6**	**-1.2**	**-1.1**	**-1.6**	**-1.3**	**-1.4**	**-1.1**	**-0.8**	**-1.2**	**-1.5**	**-1.8**	**-1.9**	**-1.8**	**-1.80**
**p-value**	**0.01**	**0.26**	**0.6**	**0.62**	**0.67**	**0.57**	**0.24**	**0.26**	**0.1**	**0.2**	**0.18**	**0.29**	**0.43**	**0.23**	**0.15**	**0.07**	**0.06**	**0.07**	**0.07**

Dynamics

Studies	Order	OR	Z-scoı	p-value
Adinortey 2011	1	**0.07**	**-2.5**	**0**
Allison 1961	2	**0.28**	**-1.1**	**0.3**
Awah 2012	3	**0.73**	**-0.5**	**0.6**
Bienzle 1981	4	**0.85**	**-0.5**	**0.6**
Brabin 1990	5	**0.88**	**-0.4**	**0.7**
Brabin 1990a	6	**0.86**	**-0.6**	**0.6**
Domarle 1999	7	**0.72**	**-1.2**	**0.2**
Enevold 2008	8	**0.75**	**-1.1**	**0.3**
Gilles 1967	9	**0.67**	**-1.6**	**0.1**
Jalloh 2004	10	**0.75**	**-1.3**	**0.2**
Jalloh 2004a	11	**0.76**	**-1.4**	**0.2**
Kruatrachue 1962	12	**0.82**	**-1.1**	**0.3**
Kruatrachue 1966	13	**0.59**	**-1.5**	**0.4**
Ruwende 1995	14	**0.82**	**-1.2**	**0.2**
Ruwende 1995a	15	**0.8**	**-1.5**	**0.2**
Segeja 2008	16	**0.75**	**-1.80**	**0.1**
Segeja 2008a	17	**0.74**	**-1.9**	**0.1**
Tantular 1999	18	**0.76**	**-1.8**	**0.1**
Tantular 1999a	19	**0.77**	**-1.80**	**0.1**

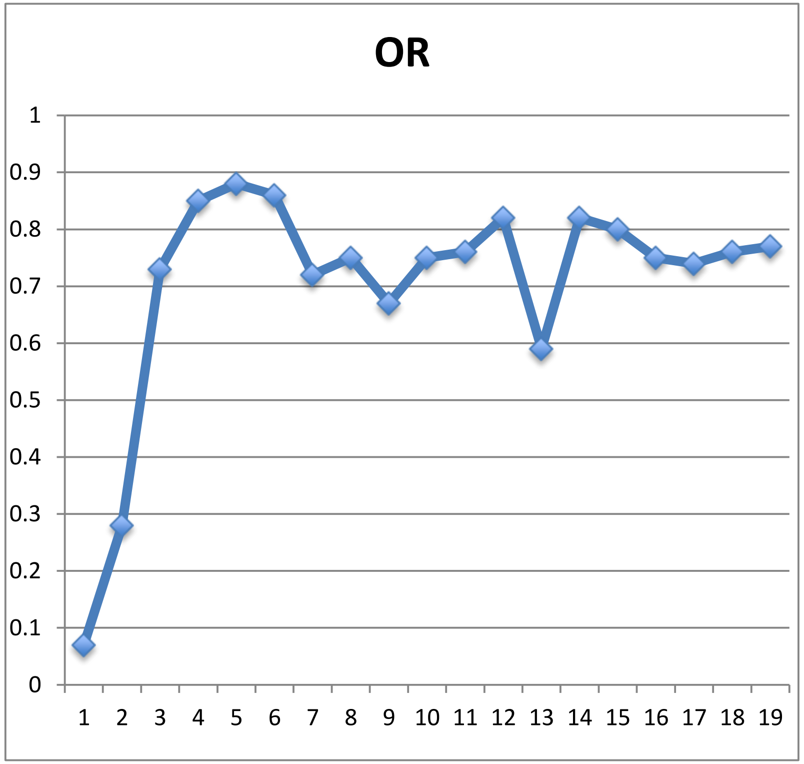

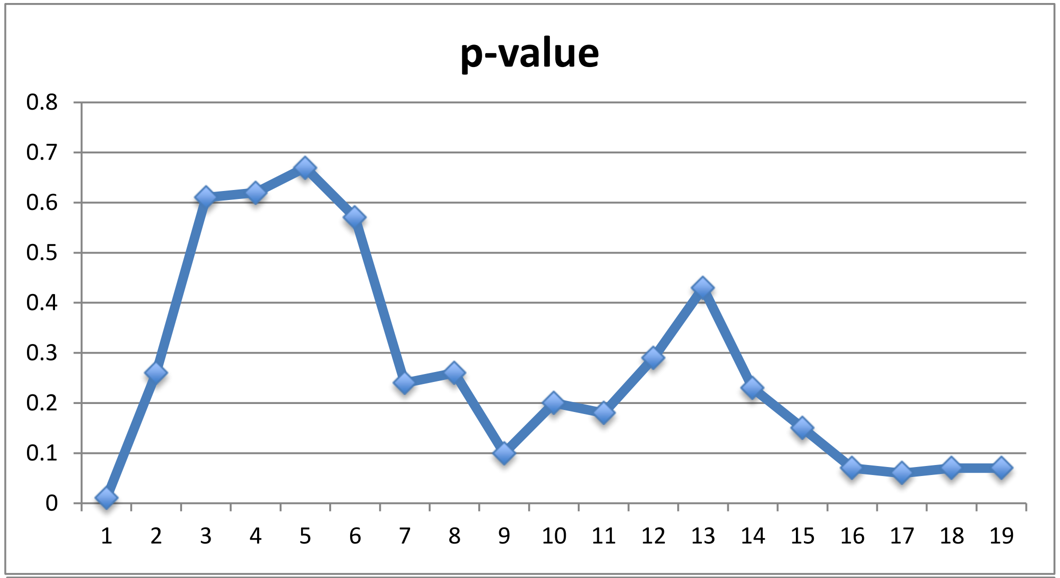
p-value
0.8
0.7
0.6
0.5
0.4
0.3
0.2
0.1
0
1 2 3 4 5 6 7 8 9 10 11 12 13 14 15 16 17 18 19

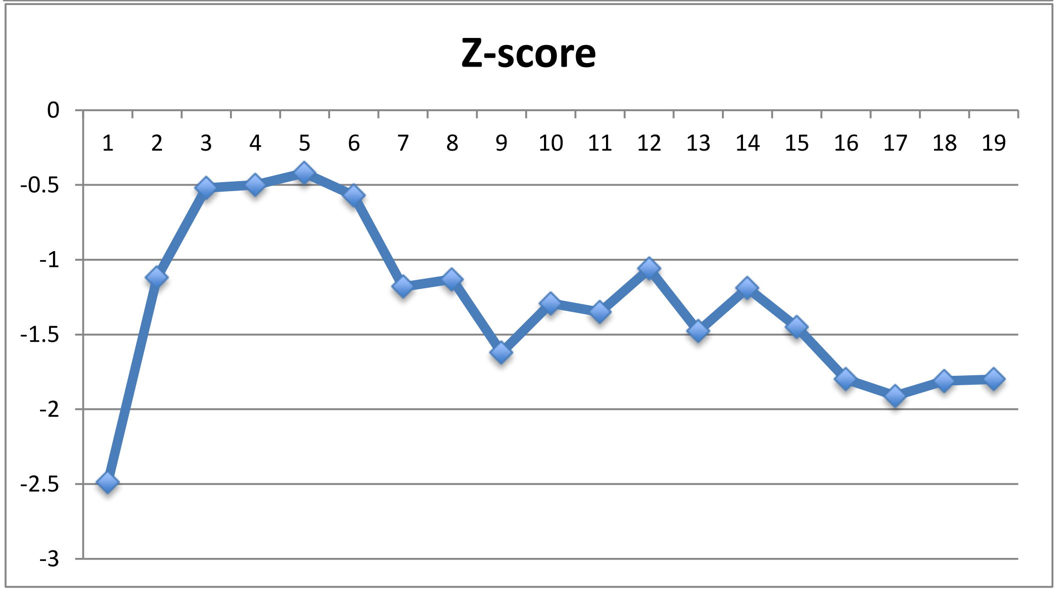
Z-score
0
-0.5
-1
-1.5
-2
-2.5
-3
1 2 3 4 5 6 7 8 9 10 11 12 13 14 15 16 17 18 19

Table 5. Sensitivity Analysis for heterozygous subgroup (Effect of individual studies)

		Effect of exclusion of one study				
**Studies**	**Order**	**OR**	**Lower CI**	**Higher CI**	**Z-score**	**p-value**
Adinortey 2011	1	0.71	0.57	0.88	3.13	0.002
Allison 1961	2	0.66	0.51	0.85	3.21	0.001
Bienzle 1981	3	0.71	0.55	0.91	2.65	0.008
Enevold 2008	4	0.70	0.56	0.87	3.26	0.001
Ruwende 1995	5	0.73	0.58	0.92	2.67	0.008
Ruwende 1995a	6	0.72	0.57	0.92	2.62	0.009
**Overall**		0.70	0.57	0.87	3.20	0.001

Table S5. Sensitivity analysis for association between G6PD defeciency and hyperparasitemia (Effe

		Effect of exclusion of one study				
**Studies**	**Order**	**OR**	**Lower CI**	**Higher CI**	**Z-score**	**p-value**
Gilles 1967	1	0.81	0.61	1.08	1.44	0.15
Allison 1961	2	0.82	0.60	1.11	1.30	0.19
Awah 2008	3	0.73	0.56	0.96	2.29	0.02
Myint 1997	4	0.72	0.53	0.97	2.15	0.03
Awah 2012	5	0.72	0.55	0.95	2.35	0.02
Lell 1999	6	0.68	0.51	0.92	2.51	0.01
Bienzle 1979	7	0.69	0.52	0.91	2.58	0.01
Kruatrachue 1966	8	0.70	0.54	0.92	2.53	0.01
**Overall**		0.73	0.56	0.95	2.30	0.02

Study or Subgroup	Malaria +ve Events	Malaria +ve Total	Malaria -ve Events	Malaria -ve Total	Weight	Odds Ratio M-H, Random, 95% CI
**1.15.1 Children only**						
Allison 1961	131	562	95	314	10.1%	0.70 [0.51, 0.96]
Bienzle 1981	138	461	85	241	9.9%	0.78 [0.56, 1.09]
Domarle 1999	4	39	7	16	2.6%	0.15 [0.04, 0.61]
Enevold 2008	26	159	5	37	4.1%	1.25 [0.45, 3.51]
Gilles 1967	6	100	34	200	4.8%	0.31 [0.13, 0.77]
Kruatrachue 1962*	21	121	38	297	7.4%	1.43 [0.80, 2.56]
Kruatrachue 1966	18	110	109	902	7.7%	1.42 [0.83, 2.45]
Ruwende 1995	41	881	39	421	8.6%	0.48 [0.30, 0.75]
Ruwende 1995a	77	499	67	292	9.5%	0.61 [0.43, 0.88]
**Subtotal (95% CI)**		**2932**		**2720**	**64.7%**	**0.72 [0.53, 0.98]**
Total events	462		479			
Heterogeneity: Tau² = 0.13; Chi² = 24.71, df = 8 (P = 0.002); I² = 68%						
Test for overall effect: Z = 2.08 (P = 0.04)						
**1.15.2 Chldren and adults**						
Adinortey 2011	1	70	12	72	1.4%	0.07 [0.01, 0.57]
Brabin 1990	2	18	8	82	2.0%	1.16 [0.22, 5.97]
Brabin 1990a	3	33	19	144	3.0%	0.66 [0.18, 2.37]
Jalloh 2004	32	222	39	361	8.2%	1.39 [0.84, 2.29]
Jalloh 2004a	8	240	17	401	5.1%	0.78 [0.33, 1.83]
Segeja 2008	3	84	21	128	3.2%	0.19 [0.05, 0.65]
Segeja 2008a	0	35	9	168	0.8%	0.24 [0.01, 4.16]
Tantular 1999	14	216	16	273	6.0%	1.11 [0.53, 2.33]
Tantular 1999a	11	205	16	277	5.6%	0.92 [0.42, 2.04]
**Subtotal (95% CI)**		**1123**		**1906**	**35.3%**	**0.72 [0.43, 1.20]**
Total events	74		157			
Heterogeneity: Tau² = 0.27; Chi² = 16.87, df = 8 (P = 0.03); I² = 53%						
Test for overall effect: Z = 1.27 (P = 0.20)						
**Total (95% CI)**		**4055**		**4626**	**100.0%**	**0.74 [0.57, 0.95]**
Total events	536		636			
Heterogeneity: Tau² = 0.15; Chi² = 42.77, df = 17 (P = 0.0005); I² = 60%						
Test for overall effect: Z = 2.32 (P = 0.02)						
Test for subgroup differences: Chi² = 0.00, df = 1 (P = 0.99), I² = 0%						

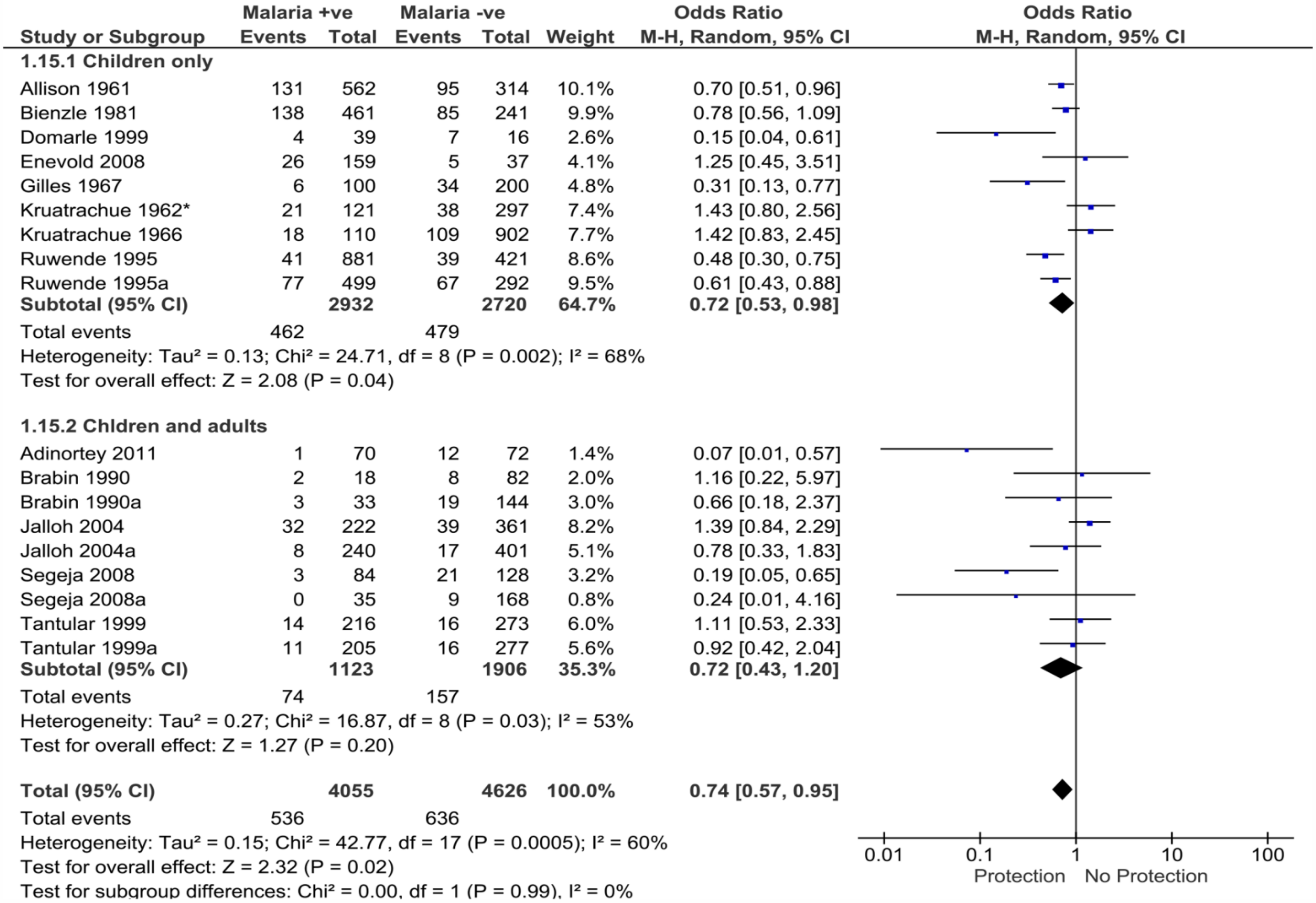

Figure S1. Meta-analysis forest plot for the association of G6PDwith malaria (subgroup analysis by age). Presented is the meta-analysis forest plot of the association of G6PD deficiency and malaria, sub-grouped into age. There was no difference in the association between G6PD deficiency and malaria for studies on children and adults. However, there was negative association for studies performed on children alone.

	Malaria +ve		Malaria -ve			Odds Ratio
**Study or Subgroup**	**Events**	**Total**	**Events**	**Total**	**Weight**	**M-H, Random, 95% CI**
**1.16.1 Males**						
Adinortey 2011	0	27	9	50	0.6%	0.08 [0.00, 1.42]
Allison 1961	58	268	47	156	6.1%	0.64 [0.41, 1.00]
Bienzle 1981	56	255	22	124	5.5%	1.30 [0.75, 2.26]
Brabin 1990	2	18	8	82	1.6%	1.16 [0.22, 5.97]
Brabin 1990a	3	33	19	144	2.3%	0.66 [0.18, 2.37]
Gilles 1967	5	55	22	102	3.1%	0.36 [0.13, 1.02]
Kruatrachue 1962*	21	121	38	297	5.3%	1.43 [0.80, 2.56]
Kruatrachue 1966	10	56	71	473	4.5%	1.23 [0.59, 2.55]
Ruwende 1995	9	461	14	239	3.8%	0.32 [0.14, 0.75]
Ruwende 1995a	32	250	28	149	5.5%	0.63 [0.36, 1.10]
**Subtotal (95% CI)**		**1544**		**1816**	**38.3%**	**0.76 [0.53, 1.09]**
Total events	196		278			
Heterogeneity: Tau² = 0.16; Chi² = 19.35, df = 9 (P = 0.02); I² = 53%						
Test for overall effect: Z = 1.48 (P = 0.14)						
**1.16.2 Females**						
Adinortey 2011	1	43	3	22	0.9%	0.15 [0.01, 1.55]
Allison 1961	73	294	48	158	6.2%	0.76 [0.49, 1.16]
Bienzle 1981	82	206	53	117	6.1%	0.80 [0.50, 1.26]
Gilles 1967	1	45	12	98	1.1%	0.16 [0.02, 1.29]
Kruatrachue 1966	8	54	38	429	4.0%	1.79 [0.79, 4.07]
Ruwende 1995	32	420	25	182	5.5%	0.52 [0.30, 0.90]
Ruwende 1995a	45	249	39	143	5.9%	0.59 [0.36, 0.96]
**Subtotal (95% CI)**		**1311**		**1149**	**29.6%**	**0.70 [0.50, 0.98]**
Total events	242		218			
Heterogeneity: Tau² = 0.08; Chi² = 10.68, df = 6 (P = 0.10); I² = 44%						
Test for overall effect: Z = 2.09 (P = 0.04)						
**1.16.3 No distinction**						
Awah 2012	30	155	23	245	5.3%	2.32 [1.29, 4.16]
Domarle 1999	4	39	7	16	2.0%	0.15 [0.04, 0.61]
Enevold 2008	26	159	5	37	3.1%	1.25 [0.45, 3.51]
Jalloh 2004	37	289	39	361	5.9%	1.21 [0.75, 1.96]
Jalloh 2004a	12	308	17	401	4.3%	0.92 [0.43, 1.95]
Segeja 2008	3	84	21	128	2.4%	0.19 [0.05, 0.65]
Segeja 2008a	0	35	9	168	0.6%	0.24 [0.01, 4.16]
Tantular 1999	14	216	16	273	4.4%	1.11 [0.53, 2.33]
Tantular 1999a	11	205	16	277	4.1%	0.92 [0.42, 2.04]
**Subtotal (95% CI)**		**1490**		**1906**	**32.2%**	**0.86 [0.52, 1.41]**
Total events	137		153			
Heterogeneity: Tau² = 0.33; Chi² = 23.45, df = 8 (P = 0.003); I² = 66%						
Test for overall effect: Z = 0.59 (P = 0.56)						
**Total (95% CI)**		**4345**		**4871**	**100.0%**	**0.78 [0.62, 0.99]**
Total events	575		649			
Heterogeneity: Tau² = 0.18; Chi² = 59.58, df = 25 (P = 0.0001); I² = 58%						
Test for overall effect: Z = 2.06 (P = 0.04)						
Test for subgroup differences: Chi² = 0.48, df = 2 (P = 0.79), I² = 0%						

Odds Ratio
M-H, Random, 95% CI

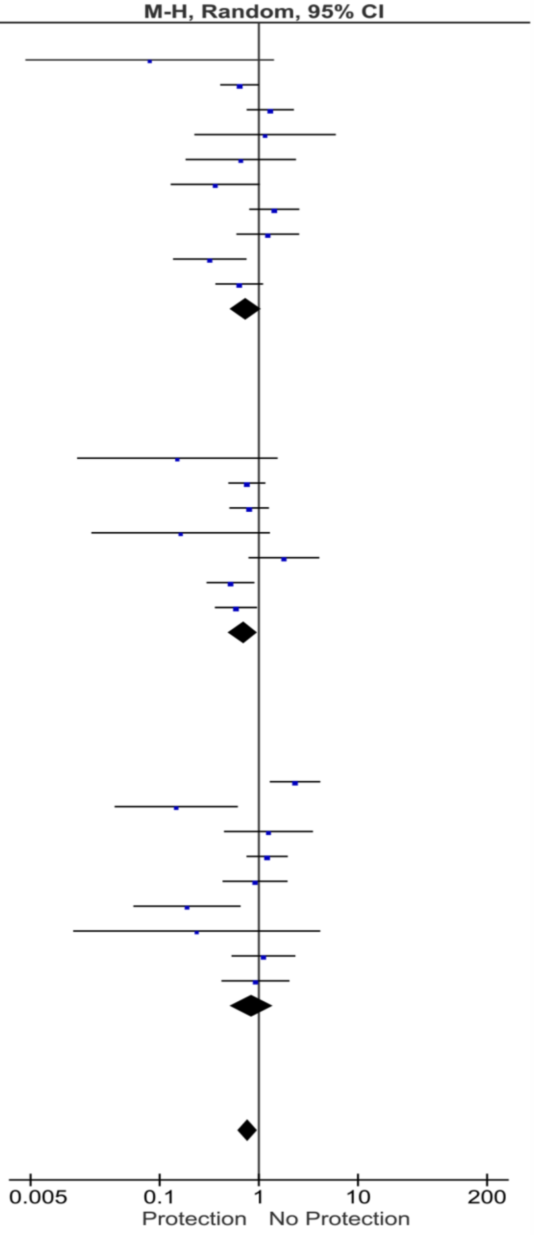

Figure S2. Meta-analysis forest plot for the association of G6PD with malaria (subgroup analysis by gender). Negative association was only found when female subjects were considered separately.

a

Study or Subgroup	Severe Malaria Events	Severe Malaria Total	Uncomplicated Malaria Events	Uncomplicated Malaria Total	Weight	Odds Ratio M-H, Random, 95% CI
Awah 2008	6	21	34	59	4.9%	0.29 [0.10, 0.86]
Gilles 1967	6	100	34	200	6.0%	0.31 [0.13, 0.77]
Guindo 2007	5	67	81	488	5.7%	0.41 [0.16, 1.04]
Allison 1961	17	112	114	450	8.9%	0.53 [0.30, 0.92]
Ruwende 1995	21	534	20	347	8.2%	0.67 [0.36, 1.25]
Guindo 2007a	40	365	340	2277	10.9%	0.70 [0.50, 0.99]
Orimadegun 2011	29	332	88	749	10.0%	0.72 [0.46, 1.12]
Myint-Oo 1995	37	243	26	140	9.0%	0.79 [0.45, 1.37]
Awah 2012	5	27	25	128	4.9%	0.94 [0.32, 2.72]
Ruwende 1995a	38	250	39	249	9.6%	0.97 [0.59, 1.57]
Lell 1999	29	99	29	98	8.4%	0.99 [0.53, 1.82]
Fanello 2008	5	21	77	681	5.1%	2.45 [0.87, 6.88]
Kruatrachue 1966	8	27	5	42	4.0%	3.12 [0.90, 10.84]
Dunyo 2011	4	18	45	1051	4.5%	6.39 [2.02, 20.18]
**Total (95% CI)**		**2216**		**6959**	**100.0%**	**0.82 [0.61, 1.11]**
Total events	250		957			

Heterogeneity: Tau² = 0.19; Chi² = 35.53, df = 13 (P = 0.0007); I² = 63%
Test for overall effect: Z = 1.27 (P = 0.20)

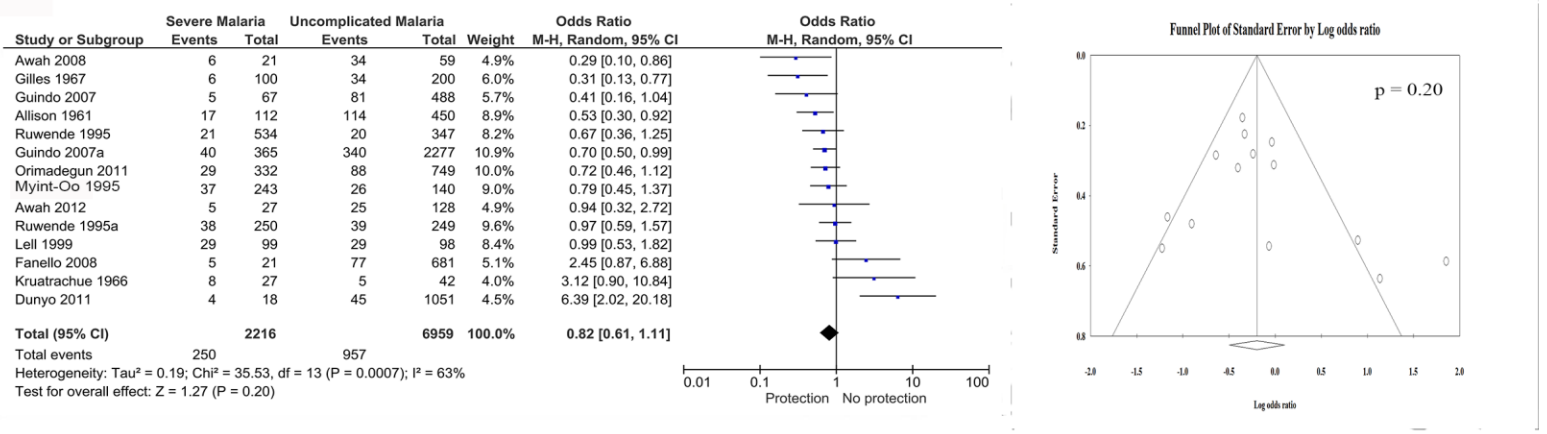

b

Study or Subgroup	Severe anaemia Events	Severe anaemia Total	Uncomplicated anaemia Events	Uncomplicated anaemia Total	Weight	Odds Ratio M-H, Random, 95% CI
Awah 2008	6	21	34	59	8.1%	0.29 [0.10, 0.86]
Guindo 2007	5	67	81	488	9.3%	0.41 [0.16, 1.04]
Ruwende 1995	6	174	20	347	9.4%	0.58 [0.23, 1.48]
Guindo 2007a	40	365	340	2277	16.0%	0.70 [0.50, 0.99]
Orimadegun 2011	29	332	88	749	15.0%	0.72 [0.46, 1.12]
Lell 1999	29	99	29	98	12.9%	0.99 [0.53, 1.82]
Ruwende 1995a	21	115	39	249	13.3%	1.20 [0.67, 2.16]
Fanello 2008	5	21	77	681	8.5%	2.45 [0.87, 6.88]
Dunyo 2011	4	18	45	1051	7.5%	6.39 [2.02, 20.18]
**Total (95% CI)**		**1212**		**5999**	**100.0%**	**0.90 [0.60, 1.37]**
Total events	145		753			

Heterogeneity: Tau² = 0.25; Chi² = 26.09, df = 8 (P = 0.001); I² = 69%
Test for overall effect: Z = 0.48 (P = 0.63)

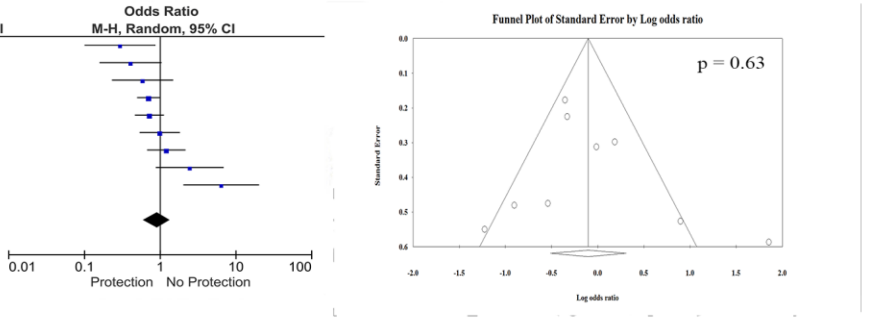

c

Study or Subgroup	Hyper-parasitemia Events	Hyper-parasitemia Total	Low-parasitemia Events	Low-parasitemia Total	Weight	Odds Ratio M-H, Fixed, 95% CI
Gilles 1967	6	100	34	200	16.4%	0.31 [0.13, 0.77]
Allison 1961	17	112	114	450	29.6%	0.53 [0.30, 0.92]
Awah 2008	0	3	7	44	0.8%	0.71 [0.03, 15.31]
Myint-Oo 1995	37	243	26	140	21.5%	0.79 [0.45, 1.37]
Awah 2012	5	27	25	128	5.4%	0.94 [0.32, 2.72]
Lell 1999	29	99	29	98	15.8%	0.99 [0.53, 1.82]
Bienzle 1979	11	35	86	302	9.4%	1.15 [0.54, 2.45]
Kruatrachue 1966	3	8	10	61	1.1%	3.06 [0.63, 14.91]
**Total (95% CI)**		**627**		**1423**	**100.0%**	**0.73 [0.56, 0.95]**
Total events	108		331			

Heterogeneity: Chi² = 10.45, df = 7 (P = 0.16); I² = 33%
Test for overall effect: Z = 2.30 (P = 0.02)

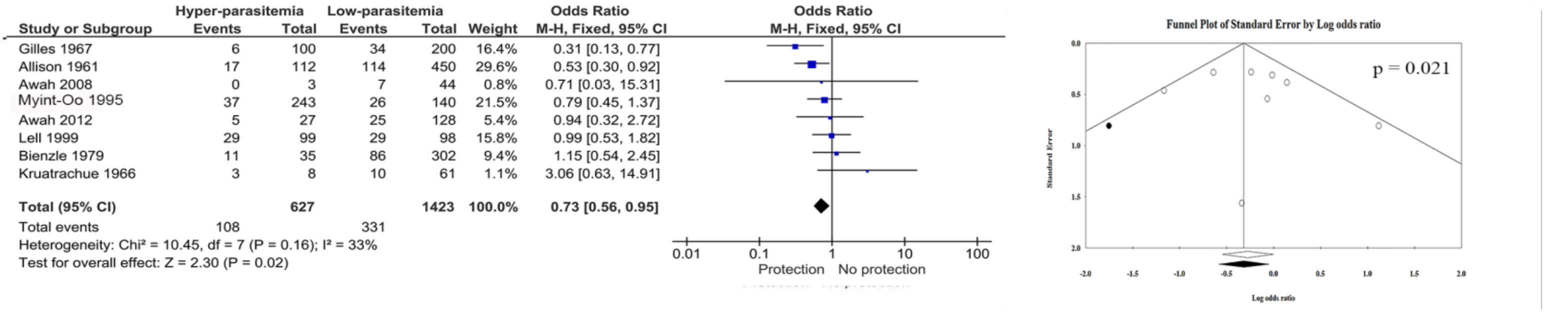

d

Study or Subgroup	Cerebral Malaria Events	Cerebral Malaria Total	Uncomplicated Events	Uncomplicated Total	Weight	Odds Ratio M-H, Fixed, 95% CI
Ruwende 1995	16	382	20	347	36.5%	0.71 [0.36, 1.40]
Ruwende 1995a	17	135	39	249	43.6%	0.78 [0.42, 1.43]
Myint-Oo 1995	7	46	26	140	19.8%	0.79 [0.32, 1.96]
**Total (95% CI)**		**563**		**736**	**100.0%**	**0.76 [0.50, 1.13]**
Total events	40		85			

Heterogeneity: Chi² = 0.04, df = 2 (P = 0.98); I² = 0%
Test for overall effect: Z = 1.35 (P = 0.18)

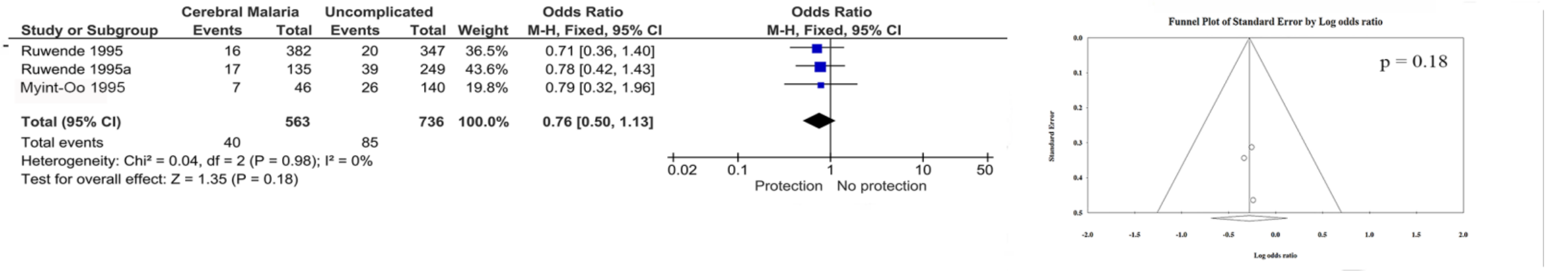

Figure S3. Association of G6PD deficiency with protection from severe malaria. (a) Meta-analysis was performed to assess association between G6PD deficiency and development of severe malaria using mild malaria patients as comparators. There was no publication bias as evident in the symmetry of the funnel plot. Generally, there was no association between G6PDdeficiency and severe malaria. When the symptoms of severe malaria were considered separately, only hyper-parasitemia shows significant association (c), but there was no association between mild malaria andsevere anemia (b)and cerebral malaria (d).

a

Study or Subgroup	Malaria +ve Events	Malaria +ve Total	Malaria -ve Events	Malaria -ve Total	Weight	Odds Ratio M-H, Fixed, 95% CI
Jalloh 2004	3	21	39	361	18.7%	1.38 [0.39, 4.88]
Jalloh 2004a	0	13	17	401	5.8%	0.81 [0.05, 14.25]
Kruatrachue 1966	5	41	109	902	42.4%	1.01 [0.39, 2.63]
Tantular 1999	0	34	16	273	18.8%	0.23 [0.01, 3.86]
Tantular 1999a	2	29	16	277	14.4%	1.21 [0.26, 5.54]
**Total (95% CI)**		**138**		**2214**	**100.0%**	**0.95 [0.50, 1.80]**
Total events	10		197			

Heterogeneity: Chi² = 1.44, df = 4 (P = 0.84); I² = 0%
Test for overall effect: Z = 0.16 (P = 0.87)

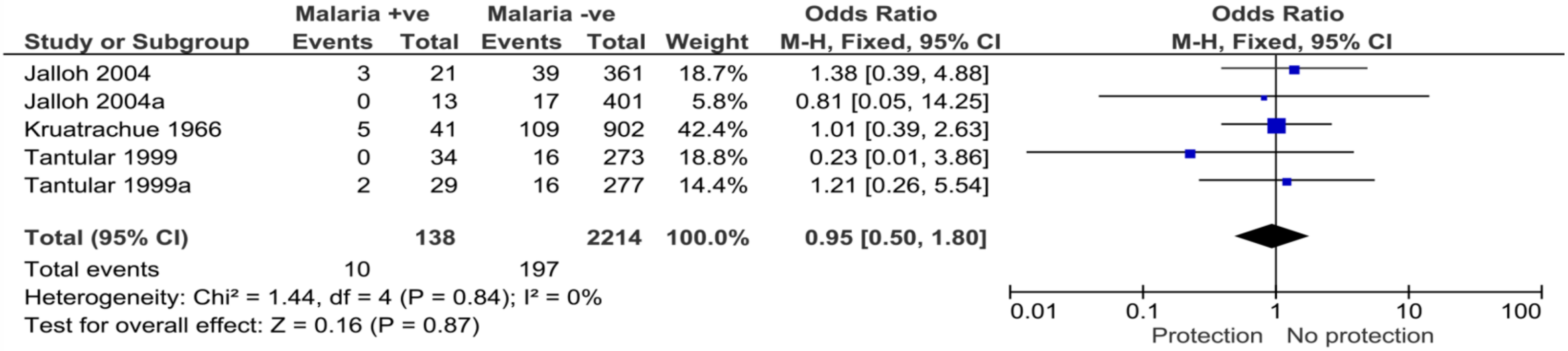

b

Study or Subgroup	Malaria +ve Events	Malaria +ve Total	Malaria -ve Events	Malaria -ve Total	Weight	Odds Ratio M-H, Random, 95% CI
Jalloh 2004	1	5	39	361	19.5%	2.06 [0.22, 18.94]
Jalloh 2004a	2	14	17	401	23.4%	3.76 [0.78, 18.16]
Nkuo-Akenji 2004	58	150	51	70	28.3%	0.23 [0.13, 0.44]
Tantular 1999	0	1	16	273	14.0%	5.20 [0.20, 132.70]
Tantular 1999a	0	2	16	277	14.8%	3.17 [0.15, 68.75]
**Total (95% CI)**		**172**		**1382**	**100.0%**	**1.56 [0.29, 8.34]**
Total events	61		139			

Heterogeneity: Tau² = 2.49; Chi² = 16.98, df = 4 (P = 0.002); I² = 76%
Test for overall effect: Z = 0.52 (P = 0.60)

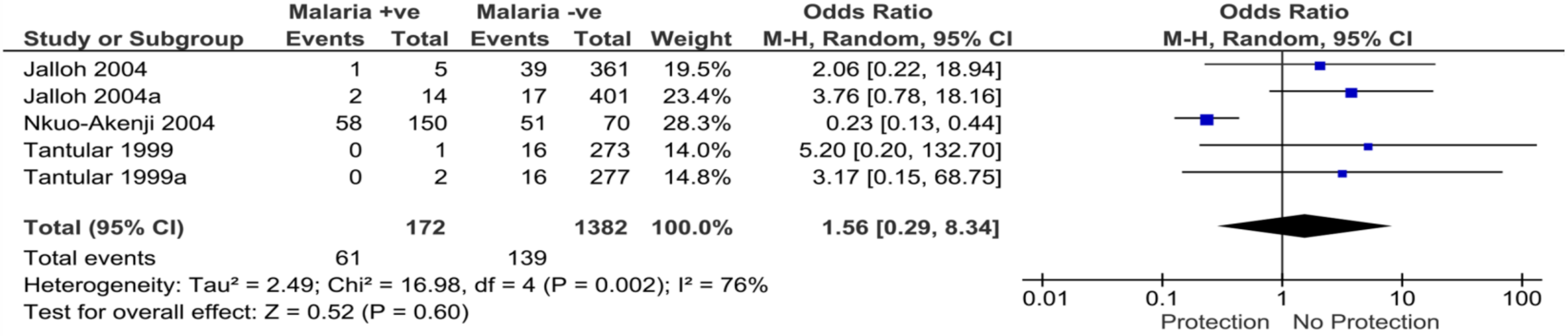

c

Study or Subgroup	Malaria +ve Events	Malaria +ve Total	Malaria -ve Events	Malaria -ve Total	Weight	Odds Ratio M-H, Fixed, 95% CI
Jalloh 2004	0	4	39	361	41.5%	0.91 [0.05, 17.17]
Jalloh 2004a	0	4	17	401	16.6%	2.44 [0.13, 47.15]
Tantular 1999a	0	8	16	277	41.9%	0.93 [0.05, 16.86]
**Total (95% CI)**		**16**		**1039**	**100.0%**	**1.17 [0.22, 6.35]**
Total events	0		72			

Heterogeneity: Chi² = 0.29, df = 2 (P = 0.87); I² = 0%
Test for overall effect: Z = 0.18 (P = 0.85)

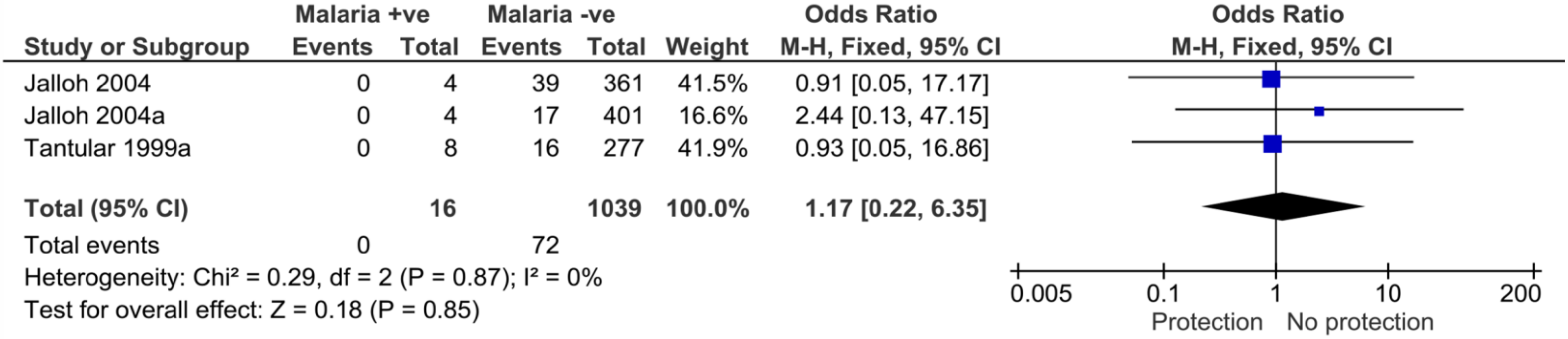

d

Study or Subgroup	Malaria +ve Events	Malaria +ve Total	Malaria -ve Events	Malaria -ve Total	Weight	Odds Ratio M-H, Fixed, 95% CI
Jalloh 2004	0	3	39	361	39.0%	1.17 [0.06, 23.00]
Jalloh 2004a	0	1	17	401	6.7%	7.32 [0.29, 186.31]
Tantular 1999	0	2	16	273	15.4%	3.12 [0.14, 67.70]
Tantular 1999a	0	6	16	277	38.9%	1.22 [0.07, 22.58]
**Total (95% CI)**		**12**		**1312**	**100.0%**	**1.90 [0.42, 8.51]**
Total events	0		88			

Heterogeneity: Chi² = 0.96, df = 3 (P = 0.81); I² = 0%
Test for overall effect: Z = 0.84 (P = 0.40)

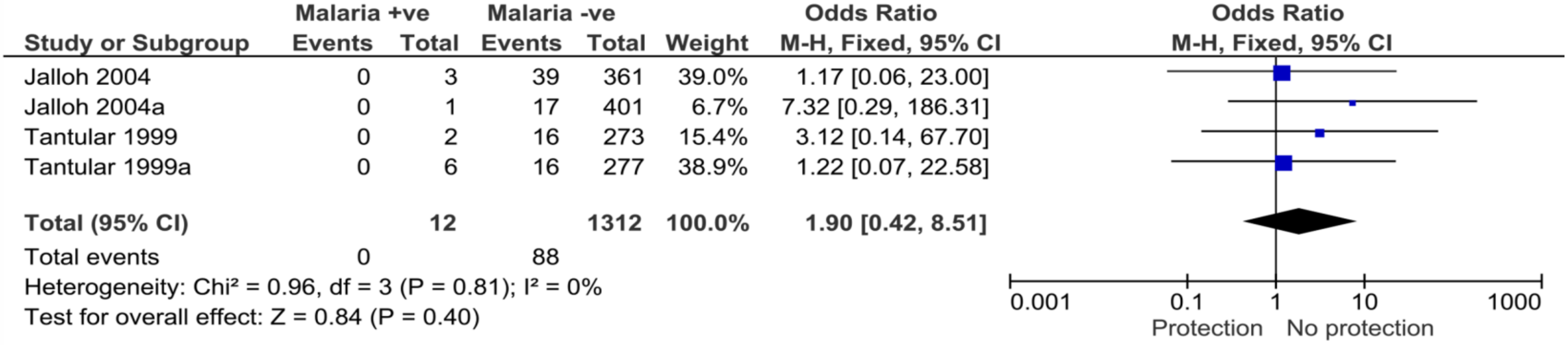

Figure S4. Meta-analysis forest plot for the association of G6PD with mixed malaria infection. The meta-analysis shows no association between G6PD and combined infection by P. falciparum and P. vivax (a), P. falciparum and P. malariae (b), P. fvivax and P. malariae (c), or P. falciparum, P. vivax and P. malariae (d)
